# Angiotensin dependent and angiotensin independent protective effects of renin-b in H9c2 cells after anoxia

**DOI:** 10.1038/s41598-020-76712-z

**Published:** 2020-11-12

**Authors:** Heike Wanka, Philipp Lutze, Doreen Staar, Katharina Bracke, Janine Golchert, Jörg Peters

**Affiliations:** Institute of Physiology, University Medicine Greifswald, Friedrich-Ludwig-Jahn-Str. 15A, 17475 Greifswald, Germany

**Keywords:** Physiology, Medical research, Molecular medicine

## Abstract

The renin-angiotensin system is known to regulate blood pressure as well as water- and electrolyte balance. An activated RAS is involved in the development of hypertension and hypertension-related organ damage. Thus, inhibitors of the RAS are protective and markedly increasing the life span of patients. In contrast, renin transcripts have been discovered encoding a cytoplasmatic renin isoform, termed renin-b, which is not harmful but may be even protective. Here we demonstrate that depletion of renin-b encoding transcripts by small interference RNA decreased ATP levels and increased basal necrosis as well as apoptosis rates. Furthermore, renin-b depletion potentiated the anoxia-induced increase of necrosis rates. Vice versa, overexpression of renin-b prevented the anoxia-induced increase of caspase-mediated apoptosis rates. Besides, cells overexpressing renin-b exhibited even reduced mitochondrial mediated apoptosis rates under anoxia, when compared with normoxic conditions, as indicated by Annexin V labeling. However, whereas the protective effect of renin-b on caspase-mediated apoptosis was completely blocked by the renin inhibitor CH732, the effect on mitochondrial-mediated apoptosis was not affected by CH732 at all. From these data we conclude that renin-b overexpression mediates cardioprotective effects under anoxia with respect to mitochondrial induced apoptosis angiotensin-independently, but with respect to caspase induced apoptosis likely in an angiotensin-dependent manner.

## Introduction

The circulating renin-angiotensin system (RAS) is well known to regulate blood pressure as well as water- and electrolyte homeostasis. However, its effector peptide, angiotensin (ANG) II, also enhances oxidative stress, exerts pro-inflammatory effects, and induces apoptotic and necrotic cell death. Thus, inhibitors of the RAS are used for the treatment of hypertension and cardiac failure^1^.

Besides the classical renin (renin-a) there are alternative renin transcripts, such as renin-b (previously also termed „exon(1a-9)renin") and renin-c, that have been identified in rats, mice, and human^2–4^. In mice, renin-b may be involved in blood pressure regulation^5^. In the rat, renin-b transcription is under the control of an alternative promoter located in intron 1^6^. Renin-b mRNA abundance increased markedly after myocardial infarction in vivo^7^. Due to the absence of the signal for a co-translational transport to the endoplasmic reticulum, encoded by exon1, all alternative renin transcripts are translated at free ribosomes into a truncated prorenin^2–4^. The protein, which we henceforth term cytosolic renin, is found in the cytosol as well as within mitochondria and exhibits enzymatic activity^2,8,9^.

Isolated beating hearts of transgenic rats overexpressing the coding region of renin-b (renin mRNA derived from exon2-9) were more resistant against ischemia-induced injury ex vivo and ren(2–9) transfected H9c2 cells exposed to glucose starvation are protected from necrotic cell death^10^. These cells also exhibit a switch to more aerobic glycolysis known as Warburg effect, which may be favorable under starvation conditions^11^. Furthermore, overexpression of ren(2–9) in H9c2 cells abolishes the increase in apoptosis rates under glucose depletion combined with hypoxia (OGD) by preserving the mitochondrial membrane potential (MMP) as well as ATP production and by preventing accumulation of reactive oxygen species (ROS)^12^. Under glucose depletion the effects of ren(2–9) overexpression were not dependent on ANG generation, since a renin specific inhibitor did not block the effects observed^10^.

We here investigate the effect of overexpression of ren(2–9) and renin siRNA depletion on anoxia-induced cell death in H9c2 cells. We also asked whether any protective effect during anoxia depends on ANG generation.

## Results

### siRNA directed to renin mRNA reduced anoxia-induced upregulation of renin-b

**Figure 1:**
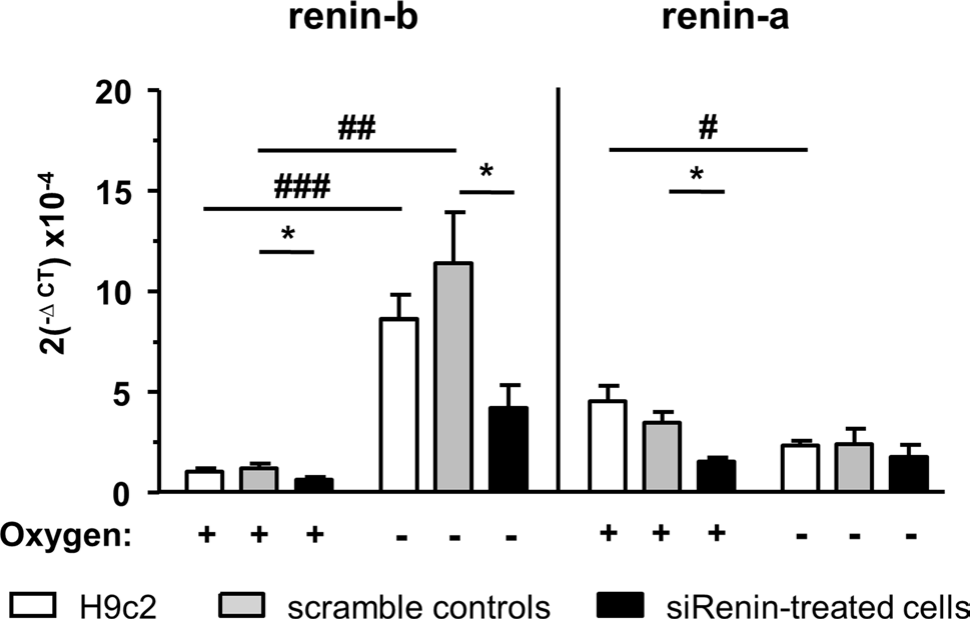
Renin mRNA abundance after anoxia and effect of renin siRNA. Renin-b and renin-a mRNA expression in H9c2 cells exposed to small interference RNA (siRNA) treatment under control and anoxic conditions (n = 6 independent experiments per group). All data represent mean ± SEM. Data are analyzed by Two-way ANOVA comparing the efficiency of renin downregulation as well as the effect of anoxia as indicated. *P < 0.05 versus scramble siRNA control as real control. ^#^*P* < 0.05 versus normoxia.

In untreated control and scrambled control H9c2 cells, endogenous renin-b transcript abundances, formerly described as ren(1a-9) renin, increased more than eightfold after anoxia. In contrast, anoxia had no effect on the expression of renin-a in scrambled control cells (Fig. 1). These data suggest that anoxia is a selective activator of renin-b expression in cardiac H9c2 cells.

Although we were not able to design specific as well as sufficiently effective siRNA for renin-b due to the fact that the transcripts are very similar, we downregulated renin with nonselective renin siRNA nevertheless, demanding that any effects of renin-b should be suppressed by siRNA. Renin siRNA markedly reduced basal and anoxia-induced renin-b expression by more than 62 ± 7% and 53 ± 4%, respectively However, renin siRNA efficiently reduced renin-a transcript abundance by 61 ± 8% under normoxic, but not under anoxic conditions (Fig. 1). Thus, unexpectedly renin-b, but not renin-a transcripts were downregulated with the siRNA under anoxia, giving us the opportunity to investigate the effect of renin-b downregulation separately.

### H9c2 cells exposed to renin-directed siRNA exhibit increased cell death

**Figure 2:**
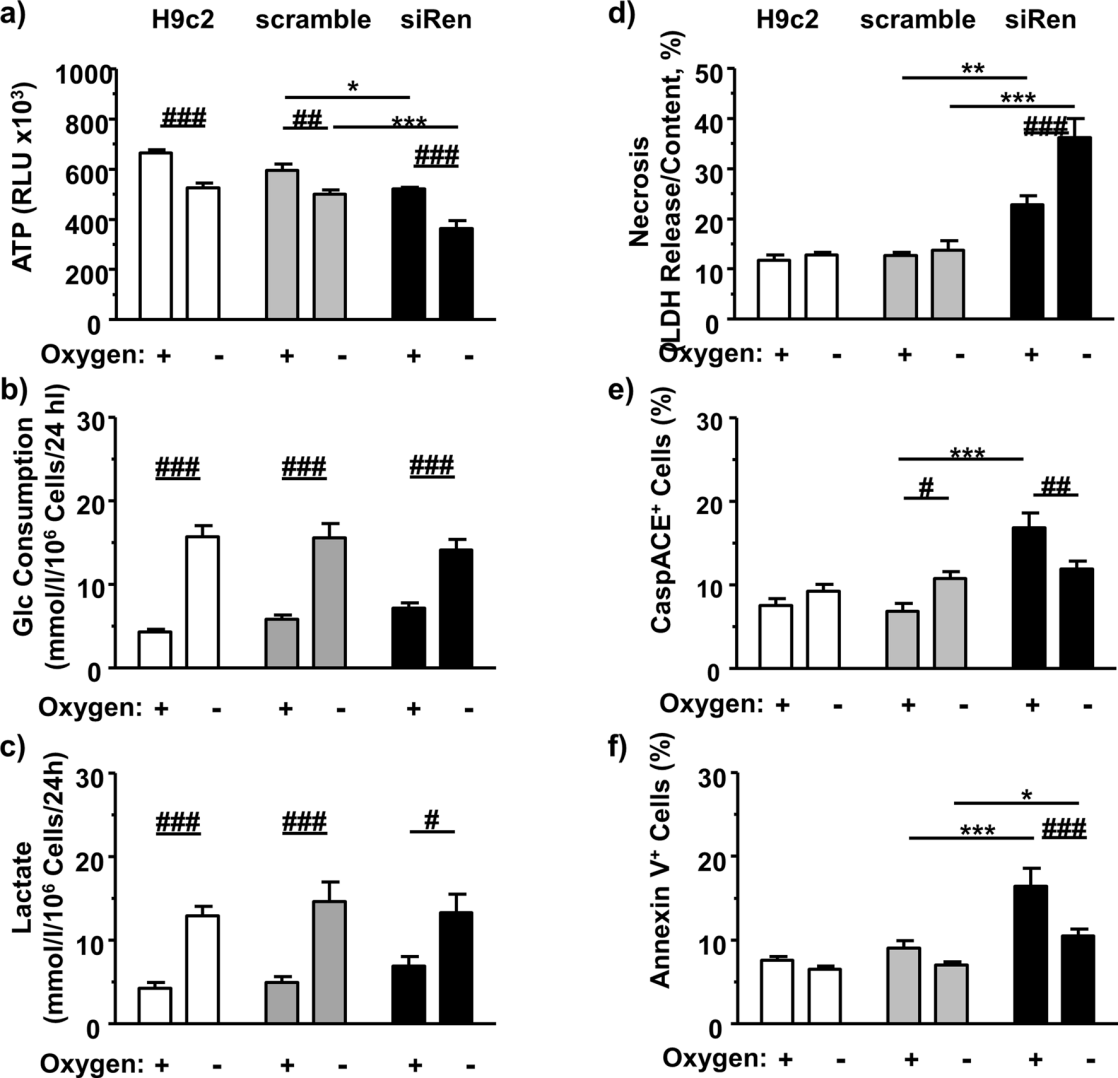
Metabolic and cell death analyses after renin downregulation. In renin downregulated H9c2 cells subjected to 24 h normoxic and anoxic conditions we measured (**a**) ATP levels (n = 8), (**b**) glucose consumption (n = 10), (**c**) extracellular lactate accumulation (n = 10), (**d**) necrosis rate (n = 8), (**e**) percentage of CaspACE-positive cells (n = 10), and (**f)** percentage of Annexin V (n = 8) (each independent experiments per group). All data represent mean ± SEM. Data of untreated control H9c2 are analyzed by t-test. Comparison of data for scramble control and siRNA-treated cells are analyzed by Two-way ANOVA comparing the effects of renin downregulation as well as the effect of anoxia as indicated. *P < 0.05 versus scramble siRNA control as real control. ^#^*P* < 0.05 versus normoxia.

To assess efficacy of anoxia, we first recorded the ATP levels as well as the glucose consumption and lactate accumulation rates (Fig. 2). There was a significant decrease of ATP levels in anoxia-exposed cells of the H9c2 control, the scramble group and the renin siRNA treated group (siRenin) (Fig. 2a). As expected, anoxia induced a significant increase of both glucose consumption and extracellular lactate accumulation. However, these changes were independent from siRNA treatment (Fig. 2b,c).

Because ren(2–9) had beneficial effects on survival of cardiac H9c2 cells exposed to glucose depletion^10^, we examined the consequences of anoxia combined with renin downregulation on necrotic and apoptotic cell death. Anoxia alone did not change the rate of necrosis in H9c2 control and scrambled control cells during the time window investigated. In comparison, renin downregulated cells showed a significantly increased necrosis rate already under normoxic conditions (Fig. 2d). Anoxia induced an additional increase of necrosis rate compared to the scrambled control cells and the siRNA treated cells grown under normoxia.

Additionally, in scrambled control cells anoxia increased the percentage of apoptotic CaspACE-positive cells (Fig. 2e), while the percentage of Annexin V-positive cells remained unchanged (Fig. 2f). Renin downregulation caused an increase of basal apoptosis rate as reflected by the increased percentages of CaspACE- and Annexin V-positive cells. Again, this indicates a protective anti-apoptotic effect of renin-b. Under hypoxia the rate of apoptosis decreased in renin siRNA-treated cells. However, this is not unexpected and may be explained by the increased necrosis rate e.g. the failure to perform apoptosis.

### Overexpression of renin-b had no effect on the expression of other RAS-relevant transcript levels

**Figure 3:**
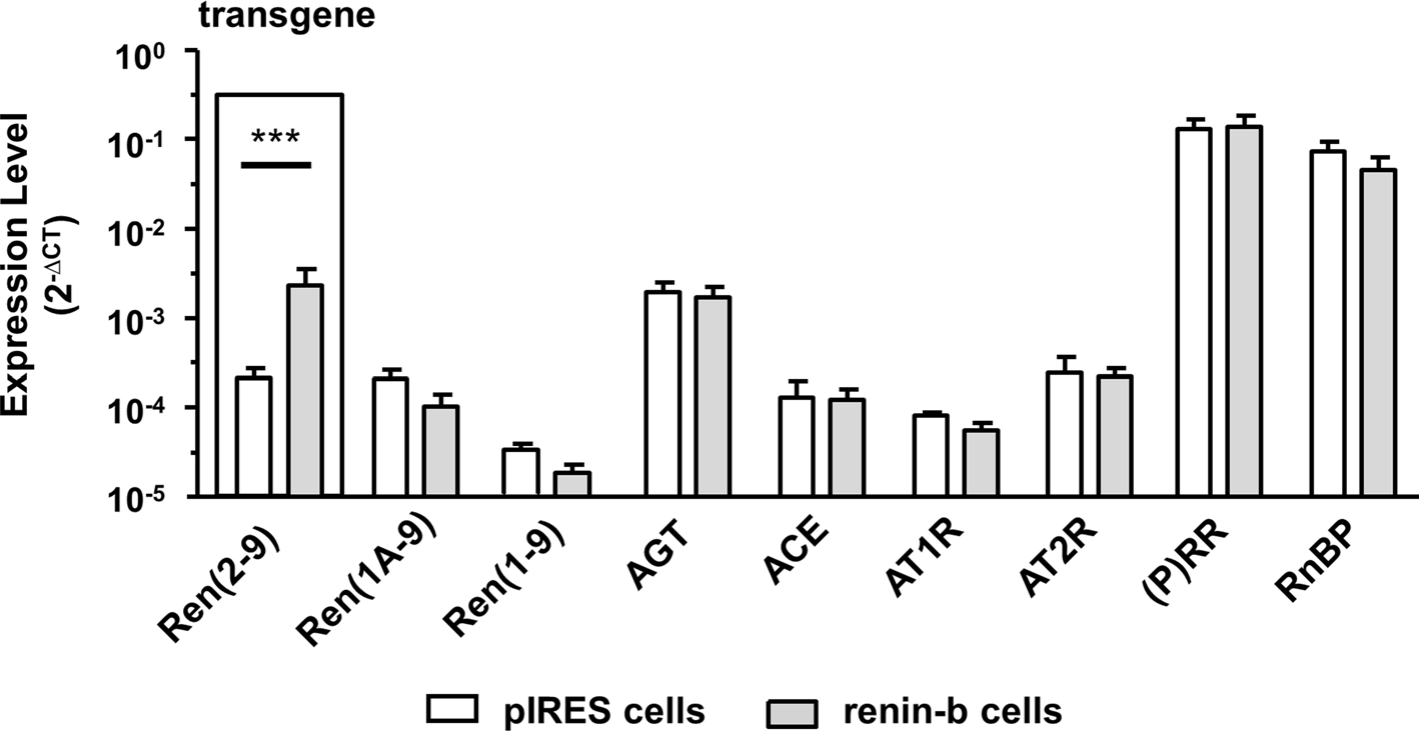
Transcript levels of RAS relevant genes. Expression of the different renin transcripts (transgenic renin(2–9), renin(1A-9) both encoding for renin-b,renin(1–9) encoding for renin-a, angiotensinogen (AGT), ACE1 and the angiotensin receptors 1 (AT1R) and 2 (AT2R), (pro)renin receptor [(P)RR], and renin binding protein (RnBP) in pIRES control cells (white columns) and renin-b transfected cells (grey columns). All data represent mean ± SEM of n = 6 experiments. Data are analyzed by Two-way ANOVA.

For this study, we used H9c2 cells transfected either with the empty- or with the ren(2–9) cDNA containing pIRES vector resulting in the generation of the pIRES control and the renin-b cell lines. Renin-b cells express a tenfold higher transgenic exon(2–9) renin transcript level encoding for renin-b (Fig. 3). In contrast, endogenous renin mRNA levels [renin(1a-9) and renin(1–9)] were unchanged in renin-b cells. Essential components of the classical RAS, such as angiotensinogen (AGT), ANG-converting enzyme (ACE), the ANG receptors AT1R and AT2R were also expressed at the same level in renin-b cells compared to pIRES cells. Additionally, the (pro)renin receptor/ATP6AP2 and the renin-binding protein/NAGE transcripts as potential interaction partners of renin were expressed at the same level as in pIRES control cells.

### The renin inhibitor CH732 modulates ATP- level, glucose consumption, lactate accumulation and annexin labeling in control-vector transfected cells

To define the protective mechanism of renin we next asked, if renin enzymatic activity i.e. intra-cytoplasmatic ANG generation may mediate the observed effects. Since it is difficult—if not impossible—to obtain correct data on ANG peptide levels in the cytosol and to differentiate between ANG produced within the cell from that taken up, we took advantage of CH732, a specific blocker of renin activity instead. CH732 is able to enter the cells and to inhibit ANG I generation by renin-a and renin-b as demonstrated previously^8,10,13^.

**Figure 4:**
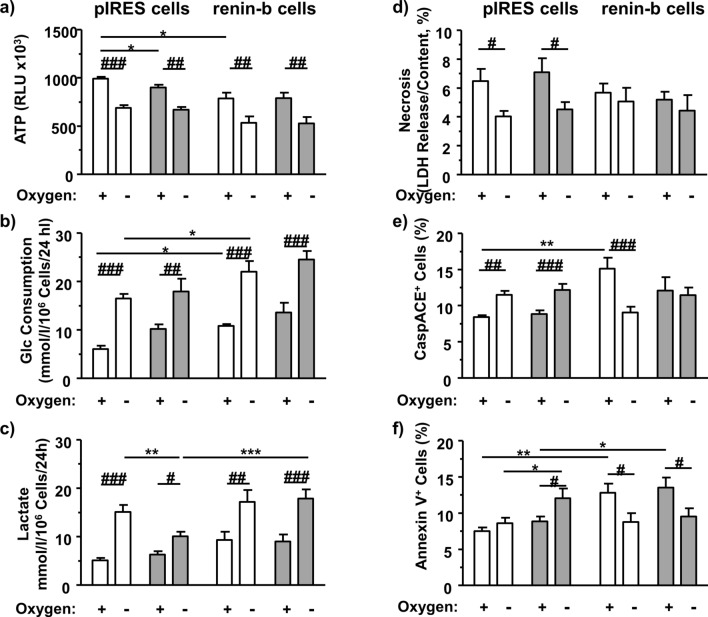
Metabolic and cell death analyses after inhibition of renin activity. H9c2 pIRES cells (control vector transfected) and ren(2–9) overexpressing H9c2 cells (renin-b cells) were subjected to 24 h normoxic and anoxic conditions without (white columns) and with renin inhibitor CH732 (grey columns). (**a**) ATP levels (n = 9), (**b**) glucose consumption (n = 8), (**c**) extracellular lactate accumulation (n = 7), (**d**) necrosis rate (n = 9), (**e**) percentage of CaspACE-positive cells (n = 7), and (**f**) percentage of Annexin V-positive cells (n = 7) were measured. All data represent mean ± SEM. Data are analyzed by Two-way ANOVA analysis comparing the effect of renin overexpression, CH732 treatment as well as the effect of anoxia as indicated. *P < 0.05 versus pIRES control. ^#^*P* < 0.05 versus normoxia.

In pIRES control vector transfected cells, CH732 slightly decreased ATP content under normoxic conditions (Fig. 4a) and lactate accumulation under anoxic condition (Fig. 4c). CH732 also increased the percentage of Annexin V-positive cells under anoxic conditions in pIRES cells (Fig. 4f).

In pIRES cells, anoxia decreased the initial rate of necrosis under the conditions applied and increased selectively the rate of CaspACE-positive cells (Fig. 4d,e). This effect was likely independent of ANG generation since it was not affected by CH732, although we cannot completely rule out non-specific effects of CH732. On the other hand, the percentage of Annexin V-positive pIRES cells was not affected by anoxia (Fig. 4f). However, CH732 induced an increase of Annexin V-positive cells in pIRES cells, which were exposed to anoxia.

#### The renin inhibitor CH732 abolishes the effect of renin-b overexpression on caspase-induced apoptosis but not on Annexin-V associated apoptosis

Renin-b overexpressing cells were initially characterized by a decreased ATP level and an increased glucose consumption rate, when compared with pIRES vector transfected cells (Fig. 4a,b). There was also a trend for increased extracellular accumulation of lactate (Fig. 4c). Anoxia, as expected, induced a decrease of ATP level and an increase of glucose consumption as well as lactate accumulation not only in pIRES cells but also in renin-b overexpressing cells (Fig. 4a–c). CH732 did not influence these metabolic parameters in renin-b cells, neither under normoxic nor under anoxic conditions.

Necrotic and apoptotic cell deaths were differentially affected (Fig. 4d–f). In renin-b cells, apoptosis rate was lower under anoxia than under normoxia. Unlike in pIRES cells, there were no differences in the necrosis rates of renin-b overexpressing cells exposed to anoxia and/or CH732 (Fig. 4d). Consistent with earlier data, renin-b overexpressing cells exhibited similar rates of necrosis when compared with pIRES control cells (Fig. 4d), while the percentages of apoptotic CaspACE-positive and Annexin V-positive cell were increased under normoxic conditions (Fig. 4e,f). The increased rate of CaspACE-induced apoptosis was abolished by CH732 (columns 1 to 5 vs columns 3 to 7) as was the anoxia induced decrease (columns 5 to 6 and columns 7 to 8) (Fig. 4e). In renin-b overexpressing cells, anoxia reduced the percentage of Annexin V-positive cells in both the control and the CH732-exposed group. Here, the anoxia effects were independent of CH732 (Fig. 4f).

## Discussion

**Figure 5:**
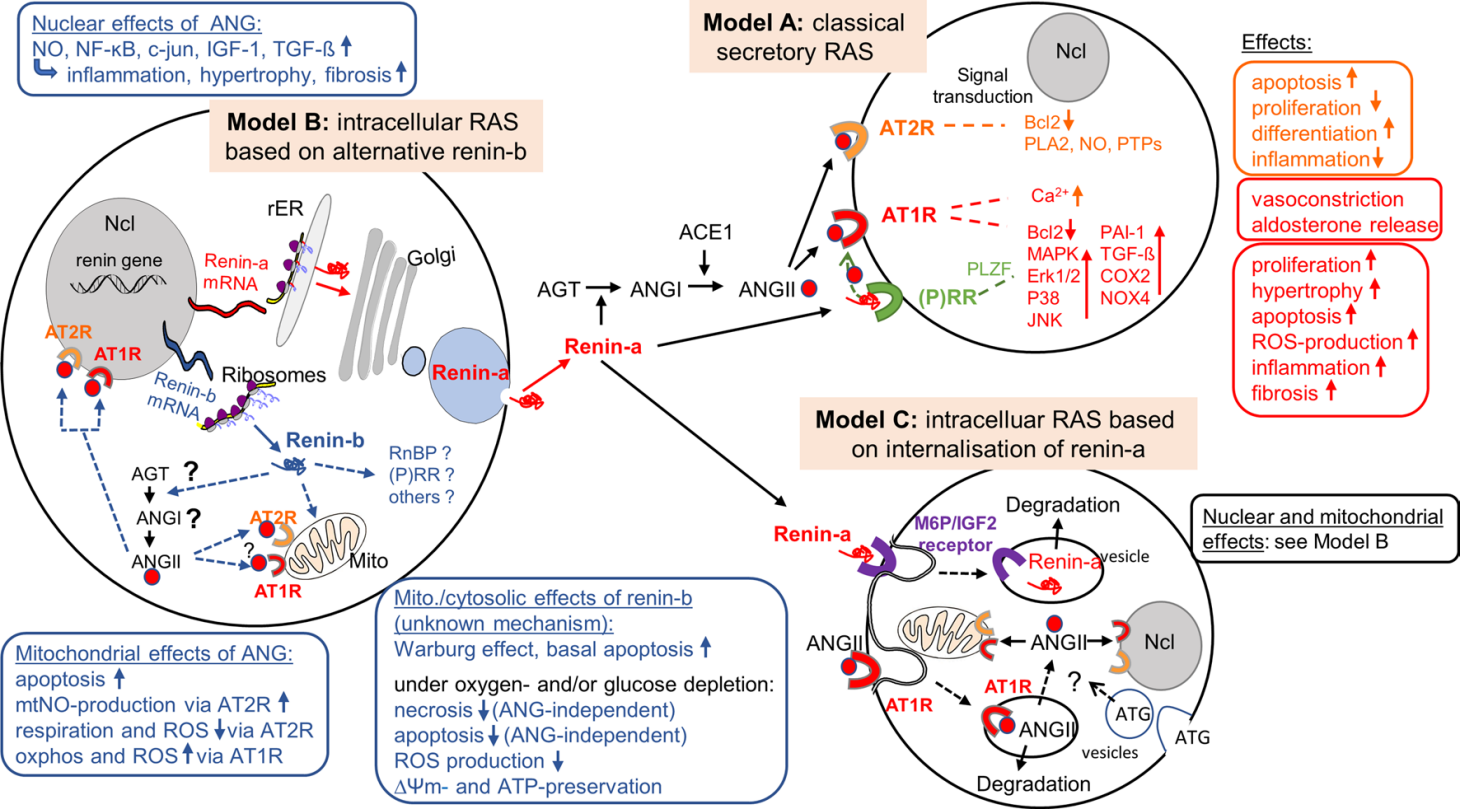
Circulating and intracellular RAS’s. Model A describes the classical secretory RAS. ANGII is generated in the circulation and activate their specific receptors located at the cell membrane followed by activation or inhibition of specific signal transduction pathways. For simplicity ACE2, other other ANG fragments (ANG1-7, ANG IV, ANG III) as well as their receptors (such as MAS) were excluded. Model B describes the molecular basis for the circulating RAS with the expression of secretory renin-a (upper part), as well as for an intracellular RAS with the expression of cytosolic renin-b (lower part). Note that renin-a and renin-b are cell type specifically differentially expressed and not necessarily coexist in the same cell. Model C describes an intracellular RAS based on uptake of renin, angiotensinogen or ANGII from the extracellular space. RAS, renin-angiotensin system; ANG, angiotensin; AT1R, angiotensin receptor type 1; AT2R, angiotensin receptor type 2; AOGEN, angiotensinogen; RnBP, renin binding protein (N-acetyl-glucosamine epimerase); (P)RR, (pro)renin receptor (ATP6AP2); NO, nitric oxide; NF-ĸB, nuclear factor kappa-light-chain-enhancer of activated B cells; c-jun, cellular jun protooncogene; IGF1, insulin like growth factor 1; TGFß, transforming growth factor ß; Ncl, nucleus; Mito, mitochondrium; rER, rough endoplasmic reticulum; mtNO, mitochondrial NO; ROS, reactive oxygen species; oxphos, oxidative phosphorylation; ∆Ψ_m_ = mitochondrial membrane potential; ATP, adenosine triphosphate; Bcl2, B-cell lymphoma 2; PLA2, phospholipase A2; PTPs, protein tyrosin phosphatases; DAC, diacylglycerine; IP3, Inositoltrisphosphat; Ca, calcium; COX2, cyclooxygenase 2; NOX4, NADPH oxidase 4; MAPK, mitogen-activated protein kinase; Erk, extracellular signal-regulated kinase; p38, p38-mitogen-activated protein; JNK, Janus kinase; PAI-1 plasminogen activator inhibitor 1; PLZF promyelocytic leukemia zinc finger protein; M6P/IGF2 receptor, mannose-6-phosphate/insulin like growth factor 2 receptor. (For review see,^37^).

There is no doubt that local RAS exist within numerous tissues^14^ in addition to the circulating RAS (Fig. 5). These local systems may play a separate role in hypertension and hypertension-related end organ damage. Importantly, they may modulate cellular functions partly independent of the circulating RAS^15,16^. Even the existence of an intracellular RAS has been proposed (for review see:^17,18^). Various experimental approaches demonstrate that intracellular (cytosolic) ANGII is able to modulate cellular, nuclear and mitochondrial functions: (1) intra-cytosolic dialysis of ANGI or ANGII^19^ as well as renin^20^ decreased the junctional conductance between cardiac cells, and these effect were abolished by RAS inhibitors, (2) ANG II injected or dialyzed into the cytosol increased intracellular free calcium levels in the cytosol and in the nucleus in an AT1R dependent manner but in the absence of membrane-bound ANG receptors^21–23^, (3) in isolated nuclei from rat cardiomyocytes ANG II stimulated the expression of NF-$\kappa$ B through AT1 and AT2 receptors located at nuclear membranes^24^, and, (4) artificial overexpression of ANGII in the cytosol increased transcript levels of c-jun, IGF-1, and TGF-ß^25^. All these effects of intracellular ANGII are harmful, stimulating apoptosis, oxidative stress, organ damage, cardiac hypertrophy, fibrosis or inflammation^25,26,27^. On the other hand, Abadir et al.^28^ identified a functionally active mitochondrial renin-angiotensin system in various cell types. The authors demonstrated using isolated mitochondria, that the activation of mitochondrial AT2 receptors increased NO production and decreased mitochondrial respiration^28^. Since the decrease of mitochondrial respiration should be associated with reduced production of ROS, this effect may be protective. Also in neurons, an intracellular RAS targeting the nuclei is protective, counteracting the harmful effects (ROS production) of extracellular ANGII^29^. Considering this, one may want to know whether inhibition of the circulating RAS is rather antagonistic or synergistic to the inhibition of the intracellular system—and whether or not the intracellular system is targeted by some RAS-inhibitors but not by others or can be targeted specifically.

The source of ANGII is still uncertain. ANGII is internalized by the AT1R. In this instance, a fraction of internalized ANGII may be subsequently released from the vesicles and then bind to nuclei or mitochondria (Fig. 5, Model C). However, whereas internalization has been demonstrated^30^, a mechanism of how ANGII may leave the vesicles is not known so far. Also, angiotensinogen appears to be internalized^31^.

On the other hand, Singh et al. demonstrated that ANG can be generated intracellularly in the absence of extracellular components of the RAS in neonatal cardiomyocytes, and that the system can be stimulated by high glucose^32^. The hypothesis of an intracellular (cytoplasmatic) generation of ANGII (Fig. 5, Model A) is supported by the fact that (1) the effect of intracellular dialysis of renin is potentiated by concomitant application of angiotensinogen^20^, and, (2) that artificial overexpression of a mutated non-secretory angiotensinogen in the cytosol in hepatoma cells increased mitogenic activity dependent on renin as well as AT1R functions^33^. However, it still remains uncertain, how angiotensinogen can be targeted to the cytosol or to mitochondria. There is no evidence to date that a transcript exists encoding for an angiotensinogen protein, which is synthetized at free ribosomes and thus present in the cytosol. On the other hand, such transcript exists for renin. An alternative renin transcript was discovered in rats and mice^2,3^, as well as in mice overexpressing a human renin transgene^4^. These transcripts encode for a renin isoform, renin-b, that is present in the cytosol and may be cardioprotective instead of harmful under ischemia related conditions^9,10^. The expression of renin-b now provides an important necessity for intra-cytoplasmatic ANG generation (Fig. 5), but also may have angiotensin independent functions (Fig. 5, Model B) (and see below).

A major new finding of the present study is that depletion of renin-b in H9c2 cardiomyoblasts (1) decreased basal levels of ATP and exaggerated the anoxia induced decrease of ATP, (2) increased basal necrosis rate and exaggerated the anoxia-induced increase of the necrosis rate, (3) increased apoptosis rates under basal conditions, but not under anoxia. The latter may be explained by the fact that renin depleted cells exposed to anoxia were not able to induce apoptosis and die by necrosis instead. From these findings, we conclude that renin-b protects cardiac cells particularly under hypoxic conditions. This is further underlined by the fact that under anoxia there is a strong association between decreased necrosis rates in pIRES cells with selective increases of endogenous renin-b expression.

Whereas renin downregulation was altogether harmful, increasing both necrosis and apoptosis rates, renin-b overexpression rather protects from necrosis by performing apoptosis instead. Renin-b overexpression in renin-b cells exhibited a favorable shift from necrosis to apoptosis under normoxic conditions and a reduced apoptosis rate under anoxic conditions when compared with basal conditions. Thus, interestingly, renin-b cells seem more stable under anoxia when compared with normoxia. Thus, it appears that cells overexpressing renin-b are primarily prepared to deal with hypoxic conditions, like cells after ischemic preconditioning^34^. Indeed, a previous study showed that cells overexpressing renin-b exhibit a shift from oxidative phosphorylation to more aerobic glycolysis known as Warburg effect (cytosolic glycolysis despite available oxygen) under basal conditions and an enhanced mitochondrial spare capacity^11^. In accordance with this, in the present study we again observed increased glucose consumption and a slightly decreased ATP level in renin-b overexpressing cells, which could not be attributed to an increased rate of necrosis as in siRNA treated cells. These cells may have disadvantages under normoxic conditions, as seen by slightly increased apoptosis rates under renin-b overexpression. Nevertheless, transgenic rats overexpressing renin-b appear to be healthy under basal conditions^35^.

Based on the expression data we found a selective upregulation of renin-b mRNA in anoxic H9c2 cells, while the expression of renin-a was decreased tendentially. These data support previous findings by Shinohara et al.^5^ who demonstrated that expression of renin-b and renin-a in the brain occurred in opposition, where renin-b inhibits the expression of renin-a. The authors further speculated that there is a selective transcriptional regulation of the renin isoforms in response to physiological or pathophysiological cues. Our data supported this concept with respect to the regulation of renin gene expression.

The mechanism of action of renin-b is still unknown. Since renin-b is translated at free ribosomes, it cannot be targeted to the secretory or lysosomal pathways. Instead, it may act in the cytosol. Additionally, it is imported in mitochondria^2^. In this context, mitochondrial functions are altered^11^, particularly in the absence of glucose^12^. Specifically, renin-b stabilizes the mitochondrial membrane potential, prevents excess production of ROS and in the cytosol it enhances non-mitochondrial oxygen consumption^11,12^. Interestingly, as discussed above, cellular functions are also modulated when ANGII is injected intracellularly^21,22^, or when isolated mitochondria are exposed to ANGII^28,29,36–38^. The expression of renin-b now provides an important necessity for intra-cytoplasmatic ANG generation. Indeed, in the present study the renin inhibitor^8,9,13,17^ appears to prevent some consequences of renin-b overexpression on apoptosis under basal conditions and anoxia. The effect of CH732 in pIRES cells on the percentage CaspACE-positive cells under normoxic conditions and on the percentage of Annexin V-positive cells under anoxic conditions indicate an involvement of angiotensin generation. Since under anoxic conditions exclusively renin-b expression and not renin-a expression was increased, angiotensin generation was likely mediated by renin-b rather than renin-a in pIRES cells. Similarly, since in transgenic renin-b cells CH732 abolished the effect of renin-b overexpression on the percentage of CaspACE-positive cells, angiotensin generation appears to take part. Although one never can rule out nonspecific effects, here the effects of CH732 support the hypothesis of the involvement of renin enzymatic activity (e.g. if renin activity was involved, CH732 must inhibit the effects and it did). Additional experiment, such as application of ACE inhibitors or angiotensin (AT1R- and AT2R) receptor blocker or knock down of angiotensinogen, ACE or ATR’s etc. would be helpful to further support the hypothesis.

In comparison, the effect of transgenic renin-b overexpression on the rate of Annexin V-positive cells e.g. its increase under normoxic conditions and its reduction to control values under anoxic conditions was independent of angiotensin generation, since CH732 was ineffective. Although we found that AGT, ACE as well as AT1 and AT2 receptors are expressed in H9c2 cells, we do not have evidence of a cytosolic location of the encoded proteins yet. It remains obscure if and how angiotensinogen or ACE should be targeted to the cytosol or to mitochondria. In this context, ANG-independent effects need to be considered (Fig. 5, Model B), particularly with respect to mitochondrial apoptosis. Two possible interaction partners are indeed expressed in H9c2 cells, namely the (pro)renin receptor/ ATP6AP2 [(P)RR)], and the cytosolic renin binding protein (RnBP). Whether the observed effects of renin-b are mediated by these proteins needs to be further investigated. Furthermore, it remains to be investigated to which degree our present findings—observed with a gene manipulated cell line—can be transferred to cardiomyocytes in vivo.

In summary, the study supports the concept of an intra-cytosolic/mitochondrial RAS, whose initial component renin-b is upregulated during anoxia. Protective effects of renin-b, respectively ren(2–9), may be partly mediated by its enzymatic activity. Furthermore, renin-b exerts ANG-independent effects probably at the mitochondrial level.

## Methods

### Cells

The cardiac specific H9c2(2–1) cell line derived from embryonic BD1X rat heart tissue by Kimes and Brandt^39^ was obtained from American Tissue Type Collection (ATTC; Manassas, VA, USA). Cells were cultured in DMEM medium supplemented with 100 U/mL penicillin, 100 µg/mL streptomycin and 10% fetal bovine serum in 75 cm^2^ tissue culture flasks at 37 °C in a humidified atmosphere of 5% CO_2_. Media exchange was performed every 3 days and cells were sub-cultured after having reached around 80% confluence.

H9c2 cells were transfected with a pIRES vector with or without *exon(2–9)renin* cDNA as previously described^9^. Upregulation of renin-b mRNA was tenfold as determined by qRT-PCR analysis. To ensure a steady overexpression, the renin-b expressing cell line and the transfected pIRES control cells (empty vector) were cultured in the presence of 430 µg/mL G418 sulfate.

Downregulation of renin in H9c2 cells by the RNA interference method was performed using 80 nmol/L siGENOME SMART pool siRNA to renin (Dharmacon, Thermo Fisher Scientific, Schwerte, Germany) according to the manufacturer’s instructions and as previously described^10^. H9c2 cells transfected with 80 nmol/L scrambled siRNA (Dharmacon, Thermo Fisher Scientific, Schwerte, Germany) served as corresponding controls. To inhibit renin enzymatic activity in terms of ANGI generation, the renin inhibitor CH732 was used in a concentration of 10^–6^ mol/L as previously reported^10^.

For functional analyses, scrambled siRNA- and siRNA-treated H9c2 cells or pIRES control cells and renin-b cells, respectively, were seeded in 6-well or 96-well culture plates, respectively, for 1 or 3 days. They were then exposed to control conditions (normoxia) or anoxia (Anaero Pack rectangular jar, Mitsubishi Gas Chemical Company Inc, Japan; GENbox anaer, Biomerieux, France) for 24 h at 5% CO_2_ and 37 °C. In another study cells were exposed simultaneously to CH732 for 24 h at 5% CO_2_ and 37 °C. Thereafter, qRT-PCR analyses for transcript abundances, detection of cell death, and analyses of metabolic parameters were performed.

### Quantitative Real-Time (RT)-PCR

RNA was extracted using the RNeasy Mini Kit (Zymo Research, Freiburg, Germany) according to the manufacturer’s instructions. RNA concentration was determined by spectrophotometry (DS-11 + , DeNovix Inc, Wilmington, USA). High Capacity cDNA Kit (Life Technologies, Darmstadt, Germany) was used for reverse transcribing RNA to cDNA, which was stored at − 70 °C. For qRT-PCR, cDNA was diluted in nuclease-free water glucose^12^. Duplicates of 20 ng cDNA were mixed with SYBR FAST Universal 2X Master Mix containing SYBR green dye and optimized primer pairs for the different transcripts and the housekeeping gene tyrosine 3-monooxygenase/tryptophan 5-monooxygenase activation protein, zeta (YWHAZ) (Table 1). Data were analyzed using the threshold cycle number (CT) in combination with the 2^−∆CT^ method and target transcripts were normalized against *Ywhaz*.

**Table 1:** Primer sequences for detection of transcript abundances.

Transcript	Forward primer	Revers primer
Renin exon(1–9)	ATGAATTCACCCCATTCAGC	CCAGATGGGCGGGAGGAGGATG
exon(1a-9)	TGAATTTCCCCAGTCAGTGAT	GAATTCACCCCATTCAGCAC
exon(2–9)	GCTCCTGGCAGATCACCAT	CCTGGCTACAGTTCACAACGTA
AGT	TGAGTTCTGGGTGGACA	GAGGAGGCGGGTTCTTTATC
AT1R	CGGCCTTCGGATAACATGA	CCTGTCACTCCACCTCAAAACA
AT2R	CAATCTGGCTGTGGCTGACTT	TGCCACTCACAGGTCCAAAGA
ACE	GCCCACCGACTCTACAACAT	ATGGGACACTCCTCTGTTGG
(P)RR	TGGGAAGCGTTATGGAGAAG	CTTCCTCACCAGGGATGTGT
RnBP	TTGCCTTCCTCATGGGTTAC	TCAGGTAGCCAAACCATTCC
YWHAZ	CATCTGCAACGACGTACTGTCTCT	GACTGGTCCACAATTCCTTTCTTG

### Functional analyses

Consequences of renin downregulation and CH732 treatment as well as anoxia on necrosis and apoptosis rates were analyzed by the Cytotoxicity Detection Kit (LDH) (Roche Applied Science, Germany) or by flow cytometry as previously described^10,12^.

### Necrotic cell death

For determination of necrosis, 1 × 10^4^ cells in 100 µL medium were seeded in 96-well plates as sixfold attempt. Three wells were used for the detection of spontaneous lactate dehydrogenase (LDH) release and the other 3 wells for the determination of the total cellular LDH content. After a 72 h growing phase at 37 °C in a CO_2_ incubator and the incubation under anoxia for another 24 h, necrosis rate was analyzed using the Cytotoxicity Detection Kit (LDH) (Roche Applied Science, Germany) as previously described^9,10^. For the detection of spontaneous LDH release, 100 µL of medium were added to 3 wells of the seeded cells, whereas in the other 3 wells 100 µl of 1% triton X-100 solution were given for 1 h to lyse the cells and record total LDH activity. Then, 100 µL of each well were transferred to another 96-well plate and incubated with 100 µL of the assay solution for 10 min in the dark. After measurements of absorbance at 490 nm, the necrosis rate was calculated by normalizing the amount of released LDH to the LDH content of the cells.

### Apoptotic cell death

For determination of apoptosis using FACS analysis^10,12^, control and treated H9c2 cells were detached from the 6-well plates by trypsin/EDTA treatment. Supernatants containing floating cells were collected and reunited with detached adherent cells. After centrifugation, cell pellets were re-suspended in culture medium. Cells were counted and apoptosis was determined by Annexin V (BD Pharmingen, Heidelberg, Germany) and CaspACE FITC-VAD-FMK (Promega, Mannheim, Germany) labeling according to the manufacturers’ instructions.

Briefly, 10^5^ cells were incubated with either 5 µL Annexin V-FITC dye in 100 µL Annexin binding buffer for 15 min at room temperature in the dark or with 5 µL of the 1:100 diluted CaspACE FITC-VAD-FMK in 100 µL culture medium for 20 min at 37 °C. The unbound antibodies or dyes were removed by washing the cells with 3 mL Annexin binding buffer or with FACS buffer, respectively.

Data from 5.000 cells were analyzed on a FACS Calibur flow cytometer (BD, Franklin Lakes, NJ, USA). Cell debris was excluded from the measurement by setting gates for intact and apoptotic cells. The data were analyzed by Cell Quest software (BD Biosciences, Franklin Lakes, NJ, USA).

### Imaging the ATP level

Cells (10^4^/well) were grown in 96-well plates for 72 h in a humidified atmosphere (5% CO_2_ at 37 °C) to allow attachment and adaptation. Then, cells were exposed to control or ischemic conditions for another 24 h before analyzing ATP levels. For measurements of ATP content, the CellTiter-Glo Luminescent Cell Viability Assay was used according to manufacturer’s instructions (Promega, Mannheim, Germany). This assay is based on the conversion of luciferin to oxiluciferin, pyrophosphate and light in the presence of ATP^11^. The quantity of light was measured using a microplate luminometer (Berthold, Bad Wildbach, Germany).

### Measurement of extracellular glucose and lactate levels

After 24 h incubation of the cells in DMEM medium at 37 °C, glucose and lactate levels were determined using the Biosens C line GF + analyzer (EKF Diagnostics, Germany) which is based on an enzymatic-amperometric sensor technology. Glucose and lactate levels of the medium alone were measured simultaneously to calculate glucose consumption and lactate accumulation rates^10,11^. For the measurement, 20 µl of the culture supernatants were incubated with 1 ml hemolyzing solution using prefilled safe-lock reaction cups (EKF Diagnostics, Germany).

### Statistics

Data are presented as means ± SEM. Statistical analyses were performed using t-test (H9c2 vs pIRES) or 2-way ANOVA followed by Bonferroni comparison procedure using GraphPad Prism 5.01 software. Differences were considered significant if *P* < 0.05.

## References

[1] Werner C (2008). RAS blockade with ARB and ACE inhibitors: current perspective on rationale and patient selection. Clin. Res. Cardiol..

[2] Clausmeyer S, Sturzebecher R, Peters J (1999). An alternative transcript of the rat renin gene can result in a truncated prorenin that is transported into adrenal mitochondria. Circ. Res..

[3] Lee-Kirsch MA, Gaudet F, Cardoso MC, Lindpaintner K (1999). Distinct renin isoforms generated by tissue-specific transcription initiation and alternative splicing. Circ. Res..

[4] Sinn PL, Sigmund CD (2000). Identification of three human renin mRNA isoforms from alternative tissue-specific transcriptional initiation. Physiol. Genomics.

[5] Shinohara K (2016). Selective Deletion of the brain-specific isoform of renin causes neurogenic hypertension. Hypertension.

[6] Lutze P, Wanka H, Bäumgen I, Staar D, Grunow B, Peters J (2017). An alternative promoter in intron1 of the renin gene is regulated by glucose starvation via serum response factor. Cell. Physiol. Biochem..

[7] Clausmeyer S, Reinecke A, Farrenkopf R, Unger T, Peters J (2000). Tissue-specific expression of a rat renin transcript lacking the coding sequence for the prefragment and its stimulation by myocardial infarction. Endocrinology.

[8] Peters J (1996). Presence of renin within intramitochondrial dense bodies of the rat adrenal cortex. Am. J. Physiol..

[9] Wanka H (2009). Cytosolic renin is targeted to mitochondria and induces apoptosis in H9c2 rat cardiomyoblasts. J. Cell. Mol. Med..

[10] Wanka H (2016). Anti-necrotic and cardioprotective effects of a cytosolic renin isoform under ischemia-related conditions. J. Mol. Med. (Berl).

[11] Wanka H (2018). An alternative renin isoform is cardioprotective by modulating mitochondrial metabolism. J. Cell. Mol. Med..

[12] Wanka H (2020). Non-secretory renin reduces oxidative stress and increases cardiomyoblast survival during glucose and oxygen deprivation. Sci. Rep..

[13] Peters J (1993). Increased adrenal renin in transgenic hypertensive rats, TGR(mREN2)27, and its regulation by cAMP, angiotensin II, and calcium. J. Clin. Invest..

[14] Paul M, Poyan Mehr A, Kreutz R (2006). Physiology of local renin-angiotensin systems. Physiol. Rev..

[15] Peters J (1999). Losartan and angiotensin II inhibit aldosterone production in anephric rats via different actions on the intraadrenal renin-angiotensin system. Endocrinology.

[16] Peters J (2008). Secretory and cytosolic (pro)renin in kidney, heart, and adrenal gland. J. Mol. Med. (Berl).

[17] Peters J (2013). Cytosolic (pro)renin and the matter of intracellular renin actions. Front Biosci. (Schol. Ed).

[18] Kumar R, Yong QC, Thomas CM, Baker KM (2012). Intracardiac intracellular angiotensin system in diabetes. Am. J. Physiol. Regul. Integr. Comp. Physiol..

[19] De Mello WC (1994). Is an intracellular renin-angiotensin system involved in control of cell communication in heart?. J. Cardiovasc. Pharmacol..

[20] De Mello WC (1995). Influence of intracellular renin on heart cell communication. Hypertension.

[21] Haller H, Lindschau C, Erdmann B, Quass P, Luft FC (1996). Effects of intracellular angiotensin II in vascular smooth muscle cells. Circ. Res..

[22] De Mello WC (1998). Intracellular angiotensin II regulates the inward calcium current in cardiac myocytes. Hypertension.

[23] Eto K, Ohya Y, Nakamura Y, Abe I, Iida M (2002). Intracellular angiotensin II stimulates voltage-operated Ca(2+) channels in arterial myocytes. Hypertension.

[24] Tadevosyan A (2010). Nuclear-delimited angiotensin receptor-mediated signaling regulates cardiomyocyte gene expression. J. Biol. Chem..

[25] Baker KM (2004). Evidence of a novel intracrine mechanism in angiotensin II-induced cardiac hypertrophy. Regul. Pept..

[26] Singh VP, Le B, Khode R, Baker KM, Kumar R (2008). Intracellular angiotensin II production in diabetic rats is correlated with cardiomyocyte apoptosis, oxidative stress, and cardiac fibrosis. Diabetes.

[27] Benigni A, Cassis P, Remuzzi G (2010). Angiotensin II revisited: new roles in inflammation, immunology and aging. EMBO Mol. Med..

[28] Abadir PM (2011). Identification and characterization of a functional mitochondrial angiotensin system. Proc. Natl. Acad. Sci. USA.

[29] Villar-Cheda B (2017). The intracellular angiotensin system buffers deleterious effects of the extracellular paracrine system. Cell Death Dis..

[30] Hunyady L, Catt KJ, Clark AJ, Gáborik Z (2000). Mechanisms and functions of AT(1) angiotensin receptor internalization. Regul. Pept..

[31] Wilson BA (2017). Angiotensinogen import in isolated proximal tubules: evidence for mitochondrial trafficking and uptake. Am. J. Physiol. Renal. Physiol..

[32] Singh VP, Le B, Bhat VB, Baker KM, Kumar R (2007). High-glucose-induced regulation of intracellular ANG II synthesis and nuclear redistribution in cardiac myocytes. Am. J. Physiol. Heart Circ. Physiol..

[33] Cook JL, Zhang Z, Re RN (2001). In vitro evidence for an intracellular site of angiotensin action. Circ. Res..

[34] Murry CE, Jennings RB, Reimer KA (1986). Preconditioning with ischemia: a delay of lethal cell injury in ischemic myocardium. Circulation.

[35] Peters J, Wanka H, Peters B, Hoffmann S (2008). A renin transcript lacking exon 1 encodes for a non-secretory intracellular renin that increases aldosterone production in transgenic rats. J. Cell Mol. Med..

[36] Re RN (2018). Role of intracellular angiotensin II. Am. J. Physiol. Heart Circ. Physiol..

[37] Escobales N, Nunez RE, Javadov S (2019). Mitochondrial angiotensin receptors and cardioprotective pathways. Am. J. Physiol. Heart. Circ. Physiol..

[38] Gwathmey TM, Alzayadneh EM, Pendergrass KD, Chappell MC (2012). Novel roles of nuclear angiotensin receptors and signaling mechanisms. Am. J. Physiol. Regul. Integr. Comp. Physiol..

[39] Kimes BW, Brandt BL (1976). Properties of a clonal muscle cell line from rat heart. Exp. Cell Res..

